# Phage-antibiotic synergy reduces *Burkholderia cenocepacia* population

**DOI:** 10.1186/s12866-022-02738-0

**Published:** 2023-01-05

**Authors:** Anna G. Mankovich, Kristen Maciel, Madison Kavanaugh, Erin Kistler, Emily Muckle, Christine L. Weingart

**Affiliations:** 1 Department of Cell and Developmental Biology, University of Illinois Urbana-Champaign, Urbana, IL USA; 2 Southeast Denver Pediatrics, Denver, CO USA; 3 Department of Pediatrics, UPMC Children’s Hospital of Pittsburgh, Pittsburgh, PA USA; 4 Department of Biology, Denison University, 100 West College Street, 43023 Granville, OH USA

**Keywords:** *Burkholderia*, *cenocepacia*, Bacteriophage, Phage-antibiotic therapy, ASMDM model

## Abstract

**Background:**

*Burkholderia cenocepacia* is an opportunistic pathogen that can cause acute and chronic infections in patients with weakened immune systems and in patients with cystic fibrosis. *B. cenocepacia* is resistant to many antibiotics making treatment challenging. Consequently, there is a critical need for alternative strategies to treat *B. cenocepacia* infections such as using bacteriophages and/or bacteriophages with subinhibitory doses of antibiotic called phage-antibiotic synergy.

**Results:**

We isolated a bacteriophage, KP1, from raw sewage that infects *B. cenocepacia*. Its morphological characteristics indicate it belongs in the family *Siphoviridae*, it has a 52 Kb ds DNA genome, and it has a narrow host range. We determined it rescued infections in *Lemna minor* (duckweed) and moderately reduced bacterial populations in our artificial sputum medium model.

**Conclusion:**

These results suggest that KP1 phage alone in the duckweed model or in combination with antibiotics in the ASMDM model improves the efficacy of reducing *B. cenocepacia* populations.

## Background

The *Burkholderia cepacia* complex (Bcc) is a group of Gram-negative opportunistic bacterial pathogens that can infect some immunocompromised individuals, including patients with cystic fibrosis (CF) and chronic granulomatous disease [1]. Currently, there are 22 species that comprise the Bcc [1–3]. Among them, *Burkholderia cenocepacia* and *Burkholderia multivorans* are responsible for causing infections in CF patients, accounting for ∼85–97% of infections, respectively [4, 5]. In some CF patients, these infections can lead to “cepacia syndrome,” which is an invasive pneumonia that can result in death [6].

Eliminating infections caused by *B. cenocepacia* is challenging due to its resistance to antibiotics, special virulence factors, and the ability to form biofilms [7]. Consequently, alternative treatment strategies, such as using bacteriophages, are being investigated. Phages provide important benefits as a treatment; they can lyse specific bacterial cells while leaving the normal microflora unharmed and they can replicate in the host allowing the number of phages to increase over time [8]. Although phage therapy was used in the 1930s, it fell by the wayside after the discovery of antibiotics [9]. With the emergence of multidrug-resistant bacteria, including *B. cenocepacia*, there has been renewed interest in phage therapy [10].

Phage therapy is effective at treating Bcc infections in vivo in both mouse and *Galleria mellonella* models [11–13]. More recently though, the idea of combining phage with sublethal doses of antibiotics (called phage-antibiotic synergy or PAS) has been gaining traction as an effective treatment against some bacterial infections [14–16]. In the case of *B. cenocepacia*, PAS is effective with the antibiotics meropenem, ciprofloxacin, and tetracycline [14]. In addition to enhanced *B. cenocepacia* killing, these PAS treatments caused an alteration in cellular morphology, increased plaque diameters, and increased phage titers. This could indicate that the presence of antibiotic causes an increase in phage production or activity, although the exact mechanisms of PAS are still unknown [14].

Overall, the goal of this research was to isolate and characterize a *B. cenocepacia* specific lytic bacteriophage and examine its potential in phage therapy and phage-antibiotic synergy as a treatment for *B. cenocepacia* in CF patients. Our results suggest, KP1 phage has potential to be an effective therapeutic, as it meets several desirable characteristics for phage therapy.

## Methods

### Bacterial strains and culture conditions

*Burkholderia cenocepacia* K56-2 was maintained in Luria-Bertani (LB) broth (1% tryptone, 0.5% yeast extract, and 1% NaCl) with shaking (120 rpm) at 37°C or on LB + 1.5% agar plates at 37°C unless otherwise noted. The *Burkholderia cepacia* complex species *B. stabilis* 14294, *B. ambifaria* AMMD, *B. dolosa* AU0645, *B. cenocepacia* J2315, *B. anthina* AU1293, *B. seminalis* AU0475, *B. metallica* AU0553, *B. diffusa* AU1075, *B. pseudomultivorans* AU3207, *B. arboris* ES263A, *B. contaminans* HI3429, *B. lata* HI4002, *B. multivorans* 17616, *B. vietnamiensis* PL259, *B. cepacia* 25416, *B. pyrrocinia* BC011 were grown in LB broth or LB agar at 37°C.

### Isolation of bacteriophages from raw sewage

Raw sewage obtained from the Newark, Ohio Treatment Plant (40 ml) was combined with 5 ml of an overnight culture of *B. cenocepacia * K56-2 and 5 ml of 10X LB broth then incubated at 37°C with shaking. After 24 h, the culture (10 ml) was centrifuged at 14,000 x *g* for 3 min, the supernatant was filter sterilized (Fisherbrand 0.45 μm nylon filter), and stored at 4°C.

For the plaque assay, the filtered sewage sample was serially diluted in phage buffer (PB) (10 mM Tris-HCl, pH 8.0; 10 mM MgCl_2_). Each dilution (100 µl) was transferred to overnight K56-2 culture grown in ½Luria-Bertani (½LB) broth (500 µl), vortexed briefly, and incubated for 5 min at 37°C. The mixtures were transferred to 0.75% soft agar (5 ml) and poured onto ½LB agar for overnight incubation at 22°C (i.e., room temperature), as this temperature allowed plaques to be more visible. Phage buffer and K56-2 each were added to top agar and plated onto ½LB agar as controls. All plates were in duplicate.

### Amplification of bacteriophage

For amplification in broth cultures, plaques were extracted using a sterile toothpick, placed in 0.2 ml PB, vortexed briefly, and transferred to 25 ml of an overnight culture of K56-2 in ½LB and incubated overnight at 37°C with shaking. The mixture was centrifuged at 5000 x *g* for 5 min at 4°C, filter sterilized, and stored at 4°C.

To determine the appropriate amount of phage needed to achieve a web pattern for amplification using plate, the phage stock was serially diluted in PB then used in a plaque assay as described previously. The appropriate dilution that achieved webbing was added to an overnight culture of K56-2 grown in ½LB. The mixture was incubated for 5 min at 37ºC, added to top agar (5 ml) then poured onto ½LB agar plates. Plates were incubated at 37°C for 24 h. Phage buffer (5 ml) was added to each plate and stored at room temperature overnight. The fluid was aspirated from the plate, centrifuged at 5000 x *g* for 10 min at 4°C, filter sterilized (0.45 μm), and stored at 4°C.

### Determination of bacteriophage host range

The host range of the bacteriophage was determined by spotting 10 µl of phage (6 × 10^9^ PFU/ml) onto ½LB plate swabbed with an overnight culture of a *B. cepacia* complex species, *Pseudomonas aeruginosa*, or *Staphylococcus aureus*. Lytic activity was measured after overnight incubation at 30 and 37°C. Species that exhibited clearing in the spot were further examined with a plaque assay to confirm lysis from the phage.

### Visualization of bacteriophage

Virion morphology was visualized with electron microscopy at The Ohio State University Campus Microscopy & Imaging Facility. Stock phage (1 × 10^8^ PFU/ml) was centrifuged at 100,000 x *g* for 1 h. Phage pellets were suspended in 1 M ammonium acetate, negatively stained with 2% (w/v) potassium phosphotungstate solution (pH 7) and visualized with a transmission electron microscope.

### Isolation of bacteriophage DNA

Phage stock (1 ml) was transferred into four separate 15 ml conical tubes. In each tube, 20 units (10 µl for 2,000 units/ml stock solution) of RNase-free DNase 1 (New England Biolabs, Ipswich Massachusetts) was added and incubated at room temperature for 15 min, then a Norgen Phage DNA Isolation Kit (Thorold, ON, Canada) was used to isolate the DNA. The concentration of DNA was measured using the Synergy LX Multi-Mode Reader (BioTek Instruments, Inc., Winooski, Vermont) nanodrop. Phage DNA libraries were prepared using the Illumina DNA prep kit and IDT 10 bp indices, and were sequenced on an Illumina NextSeq 2000 platform (San Diego, CA), producing 2 × 151 bp reads. The total 2,743,288 read pairs were demultiplexed, quality controlled and adapter trimmed with the Illumina bcl-convert (v3.9.3). The genome was assembled with SPAdes (v3.13.0). The average fold coverage was 15,727. All sequencing and assembly procedures were performed by the Microbial Genome Sequencing Center (Pittsburgh, PA).

### One step growth curve analysis

The viral propagation characteristics of KP1 were determined following a modified protocol from Summer et al. [17]. *B. cenocepacia* K56-2 was grown in LB broth to log phase at 37°C. The phage lysate (1 ml) was added to bacterial cells (1 ml) at a multiplicity of infection (MOI) of approximately 0.1. The cells were centrifuged at 14,000 x *g* for three minutes at room temperature. The supernatant was removed, and the cells were suspended in 25 ml LB broth. The infected culture was incubated with shaking at 37°C for the 90-minute duration. At time zero, a 1 ml aliquot was removed from the culture. A volume of 50 µl was serially diluted in 450 µl PB. A 100 µl volume from each dilution was added to soft agar previously inoculated with 100 µl uninfected log phase culture and poured onto LB agar. This process was repeated every ten minutes for ninety minutes. To quantify the unadsorbed phage, or free phage, 50 µl of chloroform was added to the remaining 1 ml aliquot to lyse the bacterial cells. A volume of 100 µl from the lysate was serially diluted and added to soft agar previously inoculated with 100 µl of uninfected culture. This process was repeated every ten minutes for ninety minutes. All LB agar plates were incubated at 37°C, and plaques were quantified after overnight growth.

To determine the infected bacterial cell concentration, a 50 µl aliquot of the infected culture was serially diluted in PB, plated onto LB agar, and incubated at 37°C. After 24 h, the CFU/ml was determined. The burst size was determined by dividing the total phage by the infected cells.

### Phage rescue assay with duckweed plants

Duckweed plants were purchased from Carolina Biological Supply Company, Burlington, NC. For the sterilization of the plants and determination of the LD_50_, the procedures in Thomson and Dennis [18] were used as described. Phage rescue assays were performed using a modification of the Thomson and Dennis procedure [18]. Each well of a 96-well plate was filled with 160 µl Schenk-Hildebrandt medium supplemented with 1% w/v sucrose (SHS), one sterilized duckweed plant, and 20 µl of a K56-2 culture corresponding to 100×LD_50_ in ½LB broth. The plates were covered with sterile foil then incubated at 30°C in a sterile bag for four hours to allow infection. Phage was added (20 µl of MOI = 1) and then incubated for 96 h. Controls included uninfected plants with and without phage and infected plants without phage. Plants were identified as “alive” when more than 10% of the plant remained green after 96 h, and plants that displayed > 90% loss of green pigmentation were considered dead [18]. An ANOVA (*P* < .05) and post-hoc Tukey-Kramer HSD tests were used to analyze differences. The study of plant material complies with relevant institutional, national, and international guidelines and legislation.

### Phage-antibiotic synergy assay

To test the efficacy of phage-antibiotic synergy in the Artificial Sputum Medium model (ASMDM) [19], an overnight culture of *B. cenocepacia* K56-2 (1 ml) grown in ½LB broth (OD_600_ = ~ 1.00, approximately 1 × 10^7^ CFU/ml) was centrifuged at 20,000 x *g* for 2 min and the pellet was suspended in 1ml saline. The wash was repeated. Under a sterile hood, 500µL 60% ASMDM [19] was added to wells in a gas-permeable 24-well plate (Coy Laboratory Products, Inc., Grass Lake, MI) then 10µL of the bacterial suspension was added. Two different concentrations of trimethoprim, a high dose (8.8µL of 100 mg/ml) and a low dose (8.8µL of 1 mg/ml or 1/2 x MIC) in DMSO and/or 50 µL of 9.10 × 10^8^ KH1 phage (MOI = 17) was added to the wells. The plate was incubated at 37°C with 5% CO_2_ for 72 h. At time 0 and every 24 h, cell viability (CFU/ml) was determined with a standard plate count; the samples were diluted in saline, plated in duplicate on LB agar, and incubated for 48 h at 37°C. Every 24 h, phage viability (PFU/ml) was determined with plaque assays for wells containing KP1. A repeated measures MANOVA with follow-up contrasts analyzed differences in CFU/ml and PFU/ml.

### Minimum inhibitory concentration of antibiotics

*B. cenocepacia* K56-2 was grown overnight in Mueller Hinton Broth (MHB) then diluted to an OD_600_ of 0.1 in MHB. A volume of 50 µl bacterial cells (approximately 5 × 10^5^ CFU/ml) was delivered to wells containing serially diluted antibiotic in a 96-well SpectraPlate (PerkinElmer, Waltham, MA). The plates were incubated at 37°C, and growth was examined after 24 h. The MIC was determined to be the lowest concentration with no visible growth [20].

## Results

### Characterization of KP1 phage structure and genome

**Fig. 1:**
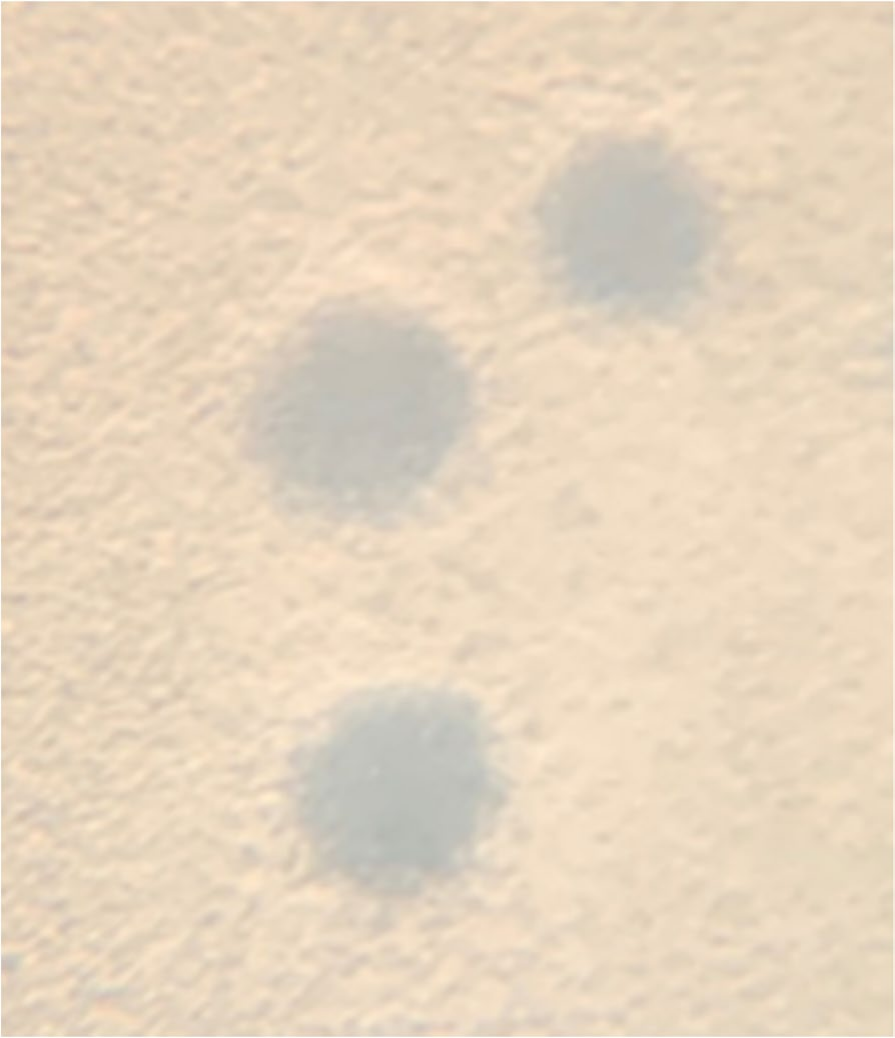
Plaque morphology of KP1 phage at 37°C. Plaques were visualized with a Zeiss Stemi 2000-C stereo microscope at 16X magnification. Scale bar represents 1 mm

**Fig. 2:**
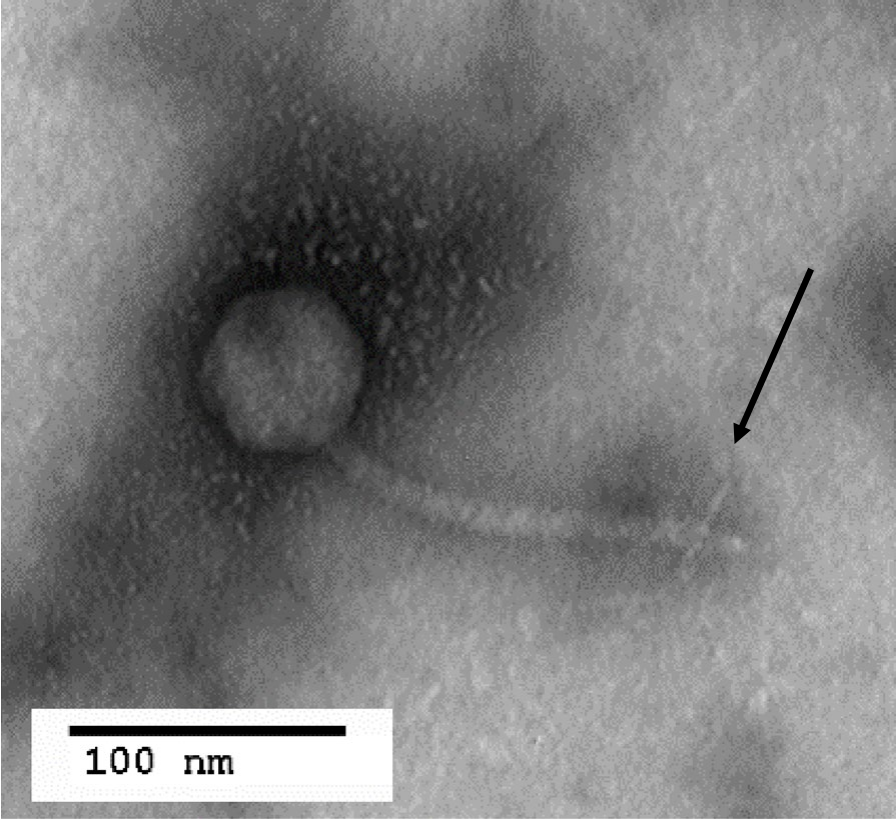
Transmission electron micrograph of phosphotungstic acid-stained KP1 phage at 120,000X magnification. Scale bar represents 100 nm. Arrow is pointing to tail fibers. Images were collected by The Ohio State University Campus Microscopy and Imaging Faci

The KP1 phage plaques were round with a uniform periphery and showed complete lysis (Fig. 1) at 30 and 37°C. The average diameter was 1 mm at 37°C and 1.5 mm at 30°C. Electron microscopy revealed the KP1 phage has an icosahedral head that measured 59 nm wide and a tail with tail fibers that measured 155 nm long (Fig. 2). KP1 phage has a double-stranded circular DNA genome of 52,676 bp. A BLASTn alignment showed our phage KP1 is most similar to the unclassified *Burkholderia* phage BcepGomr (GenBank NCBI Reference Sequence: NC_009447.1) with 89% identity over 93% of the genome. Based on the morphology and BLAST sequence comparison, the KP1 phage could be in the Class *Caudoviricetes*, Order *Caudovirales*, and family *Siphoviridae*. Currently, we are in the process of annotating the genome.

### Host range of KP1 phage

The host range was determined for the 17 *Burkholderia cepacia* complex species at 30 and 37°C using the spot assay. At both temperatures, *B*. *cenocepacia* K56-2, *B. cenocepacia* J2315, *B. dolosa* AU0645, *B. seminalis* AU0475, *B. cepacia* 25416, *B. lata* HI4002, and *B. arboris* ES263A showed an area of clearing (Table 1). The host range outside of the *Burkholderia cepacia* complex was also tested at 30 and 37°C; the phage did not infect *P. aeruginosa* BAA-81, *S. aureus* ATCC 12228, or *E. coli* B at either temperature (Table 1). Species that showed clearing in the spot assay were further examined with a plaque assay at 30 and 37°C, and only K56-2, J2315, AU0645, AU0475, ES263A and showed plaques.

**Table 1:** Host range of KP1 bacteriophage within and outside of the *B. cepacia* complex

Species	Strain	30 °C	37 °C
*B. cepacia*	25416	+	+
*B. cenocepacia*	K56-2	+	+
*B. cenocepacia*	J2315	+	+
*B. multivorans*	17616	-	-
*B. dolosa*	AU0645	+	+
*B. stabilis*	LMG 14294	-	-
*B. vietnamiensis*	PC259	-	-
*B. ambifaria*	AMMD	-	-
*B. anthina*	AU1293	-	-
*B. pyrrocinia*	BC011	-	-
*B. seminalis*	AU0475	+	+
*B. metallica*	AU0553	-	-
*B. diffusa*	AU1075	-	-
*B. pseudomultivorans*	AU3207	-	-
*B. arboris*	ES263A	+	+
*B. contaminans*	HI3429	-	-
*B. lata*	HI4002	+	+
*P. aeruginosa*	BAA-81	-	-
*S. aureus*	ATCC12228	-	-
*E. coli*	B	-	-

Data represents three independent experiments

Lytic activity was measured as follows: (-) no lysis, (+) lysis

### One step growth curve of KP1 phage

**Fig. 3:**
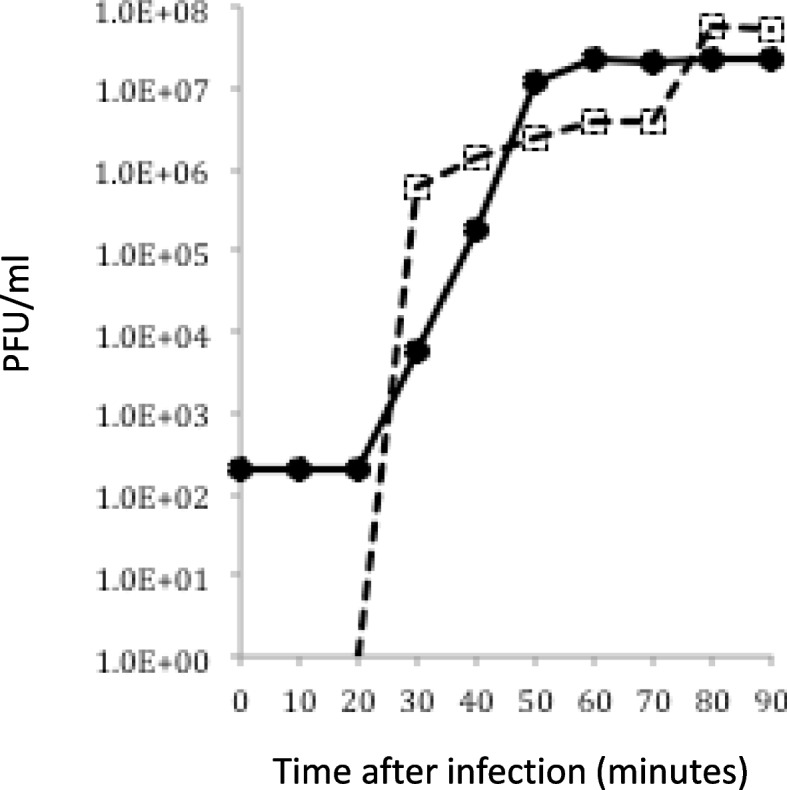
A one-step growth curve of KP1. The phage was adsorbed to log phase *B. cenocepacia* K56-2 cells at low MOI (0.073). Virion production was measured from samples taken at 10-minute intervals for 90 min to quantify intracellular (adsorbed) and free virion (unadsorbed) PFU. Unadsorbed samples are represented by squares. Adsorbed samples are represented by circles. The latent period is at 50 min. The eclipse period occurred from time 0 until 25 min when the first virion was detected in the unadsorbed samples. This is a representative of two independent experiments

A one step growth curve revealed several propagation characteristics of KP1 (Fig. 3). The average burst size was 105 PFU per cell. The eclipse period is when virus particles adsorb and transcription and translation begin within the cell. This is measured to the approximate time the first virion is detected in the chloroform treated samples, which was 25 min (Fig. 3). The latent period was observed from the time the increase in free virions was approximately 50% completed. This period was identified from time zero to approximately 50 min after infection (Fig. 3).

### Phage rescue in the duckweed infection model

**Fig. 4:**
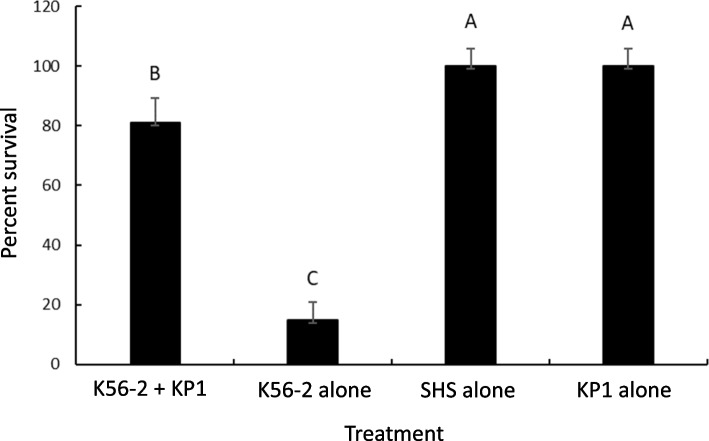
Bacteriophages rescued *B. cenocepacia* K56-2 infected duckweed plants. One plant per well containing SHS medium was treated with bacteria (20 µl of 100xLD_50_) at 30°C for four hours to establish an infection. KP-1 bacteriophages (20 µl at MOI 1 per well) were added to the wells and incubated at 30°C. Plant survival was examined after 96 h. Each experimental group had 14–18 plants and each control had eight plants per experiment. Mean ± standard error is from three independent experiments. Letters show statistically pairwise differences (ANOVA, *P* < .05; post-hoc Tukey Kramer HSD)

Phage rescue assays were performed to determine if duckweed plants could be rescued by the KP1 phage from a *B. cenocepacia* K56-2 infection. Duckweed plants treated with phage showed a significantly higher survival compared to the K56-2 control (ANOVA, *P* < .0001, Fig. 4). The phage only and medium only controls were also significantly different from the K56-2 control. Although KP1 rescued the plants from infection, the level of rescue did not reach the uninfected controls, as KP1 treatment was significantly different from them (*P* < .0019).

### Phage-antibiotic synergy on *B. cenocepacia* growth

**Fig. 5:**
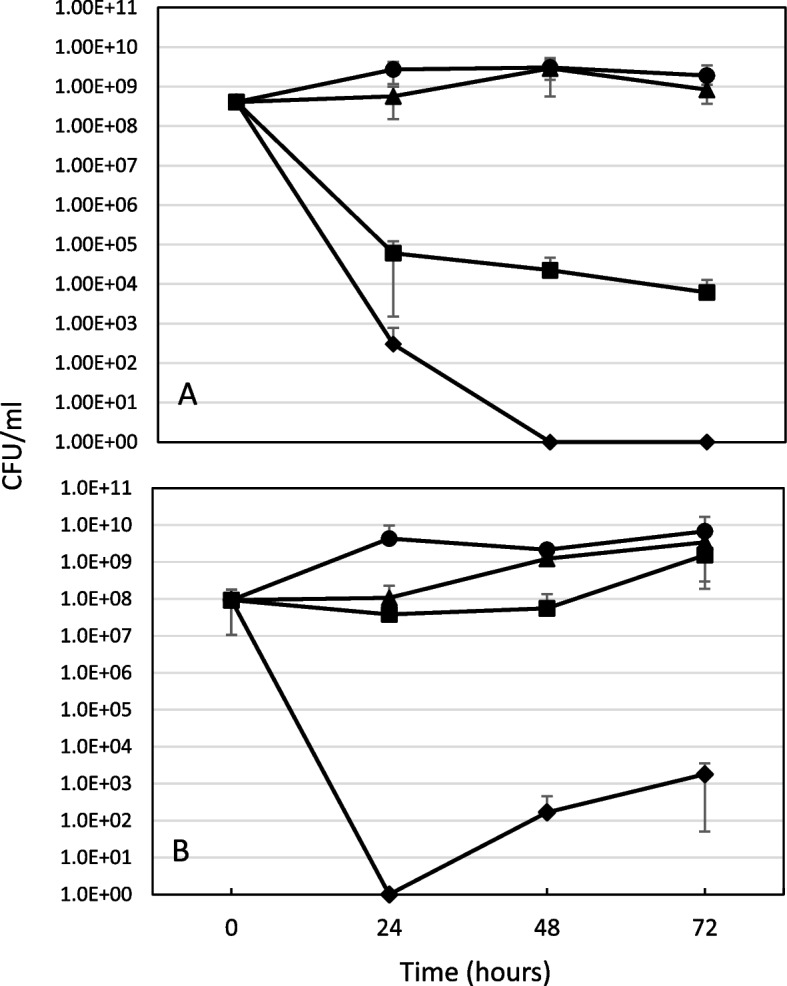
Effect of PAS on *B. cenocepacia* K56-2 growth in ASMDM over time. Bacterial cells suspended in 60% ASMDM were treated with PAS, phage, or trimethoprim at two doses. **A**) Treatment with high dose of trimethoprim (0.88 mg/well). *N* = 4. **B**) Treatment with low dose of trimethoprim (0.0088 mg/well). *N* = 3. *B. cenocepacia* K56-2 no treatment (circle), PAS treatment (diamond), trimethoprim (square), KP1 phage (triangle). MOI = 17. Values are mean ± standard deviation. Statistical analysis was performed with MANOVA, *P* < .05. The PAS treatments are significantly different from the untreated K56-2 and K56-2 treated with phage or trimethoprim

To determine the effect that phage and antibiotics have on *B. cenocepacia* growth, we used our ASMDM model that uses artificial CF sputum. For this model, bacterial cells were treated with phage and trimethoprim (an antibiotic commonly used to treat *B. cenocepacia* infections), trimethoprim at two doses, and phage alone. After 24 h the *B. cenocepacia* fell to nearly undetectable levels when treated with PAS with both high (Fig. 5A) and low dose trimethoprim (Fig. 5B). In PAS with high dose trimethoprim, *B. cenocepacia* remained eliminated after 24 h (Fig. 5A), however, in the PAS treatment with low dose trimethoprim, there was a 3-log resurgence of *B. cenocepacia* growth by 72 h after the initial treatment (Fig. 5B). When comparing phage alone to the untreated control, the greatest reduction in the amount of *B. cenocepacia* occurred 24 h after treatment with about a 1-log decrease before returning to a similar level of growth as the untreated control (Fig. 5A and B). With the high dose trimethoprim there was about a 5-log decrease in *B. cenocepacia* growth 72 h after treatment (Fig. 5A), while the low dose trimethoprim showed only a 2-log decrease in *B. cenocepacia* growth after 24 and 48 h before returning to a similar level of growth as the K56-2 control (Fig. 5B). A repeated measures MANOVA was applied to assess the effect of treatment on growth over time. There was a significant difference between the low (*P* < .0001) and high (*P* < .0001) PAS treatments when compared to all the other treatments, indicating that PAS was more effective at eliminating K56-2 compared to phage alone and antibiotic alone. High dose trimethoprim was also effective over time (*P* < .0001) compared to phage alone and antibiotic alone, however, it was not as effective as PAS (*P* < .0001), as there was a significant difference between them.

**Fig. 6:**
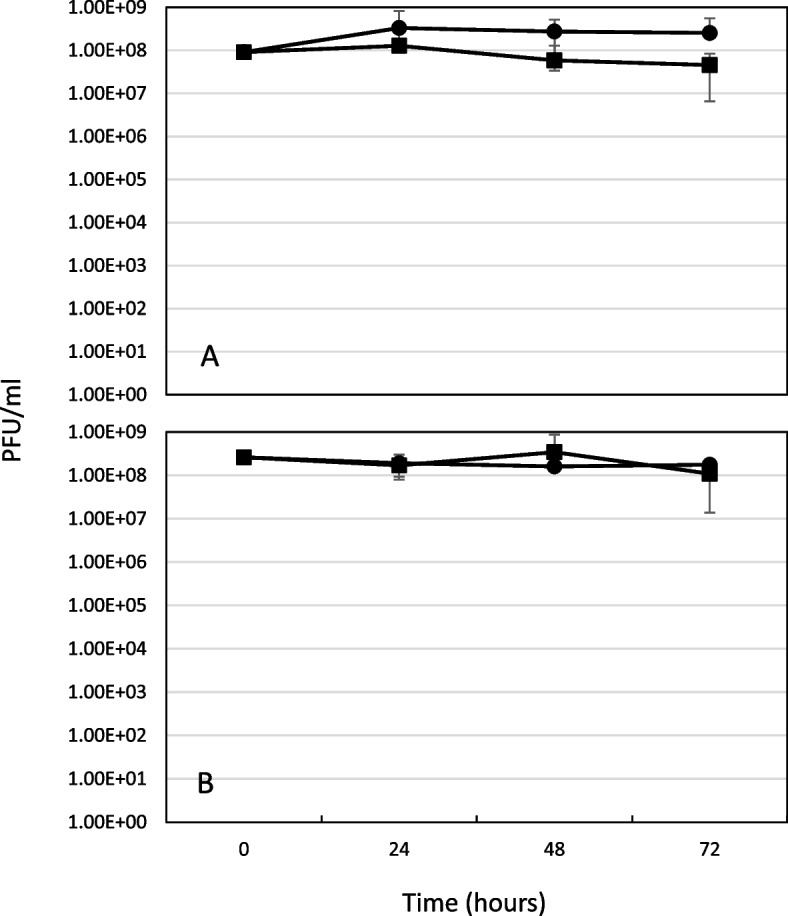
Effect of ASMDM, trimethoprim, and *B. cenocepacia* on KP1 viability. Phage viability was measured in the wells that contained phage. At 0 and every 24 h for 72 h, a plaque assay was performed to determine PFU/ml. **A**) PAS treatment with high dose of trimethoprim (0.88 mg/well). *N* = 4. **B**) Treatment with low dose of trimethoprim (0.0088 mg/well). *N* = 3. Values are mean ± standard deviation. MOI = 17. PAS treatment (circle), KP1 phage alone (square). Statistical analysis was performed with MANOVA, *P* < .05. There were no significant differences

Studies have shown that phage titers are higher and plaque diameters are larger in the presence of PAS [14, 21]. To determine if PAS affected phage titers in ASMDM, the PFU/ml was examined over time. A repeated measures MANOVA indicated there were no significant differences in the amount of phage over time between treatments (*P* > .05) (Fig. 6A and B) nor were there any observable differences in plaque sizes.

### Phage-antibiotic synergy on *B. cenocepacia* pellicle formation

**Fig. 7:**
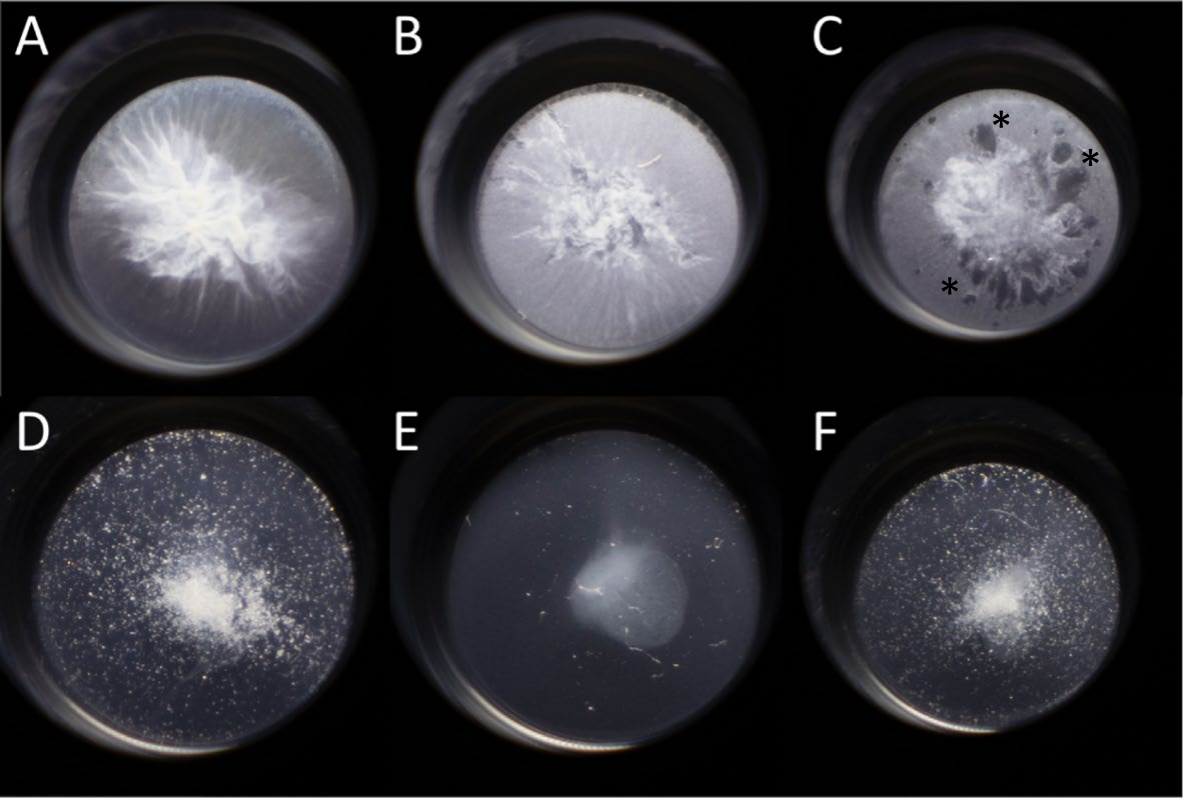
*B. cenocepacia* pellicle formation when grown in ASMDM for 72 h at 37°C, 5% CO_2_. **A**) Untreated K56-2, **B**) KP1 phage treatment, **C**) Low dose trimethoprim, **D**) High dose trimethoprim, **E**) PAS with low dose trimethoprim, and **F**) PAS with high dose trimethoprim. Asterisks in well C denote holes in the pellicle. Images collected with the assistance of Chris Faur, Denison University

Upon examination of the PAS experiments, a *B. cenocepacia* pellicle with a fibrous appearance was observed in the K56-2 untreated well, the KP1 phage treated well, and the low dose trimethoprim treated well (Fig. 7). While the phage treatment did not appear to have a large effect on pellicle formation compared to the untreated K56-2 well, the low dose trimethoprim caused a moderate disruption in the pellicle formation (Fig. 7). But, when the untreated K56-2 well was compared to the PAS with low or high dose trimethoprim treatment or high dose of trimethoprim, pellicle formation was drastically reduced, and the fibrous appearance was not detected. (Fig. 7).

### Phage-antibiotic synergy on *B. cenocepacia* phage-resistant mutant formation

**Fig. 8:**
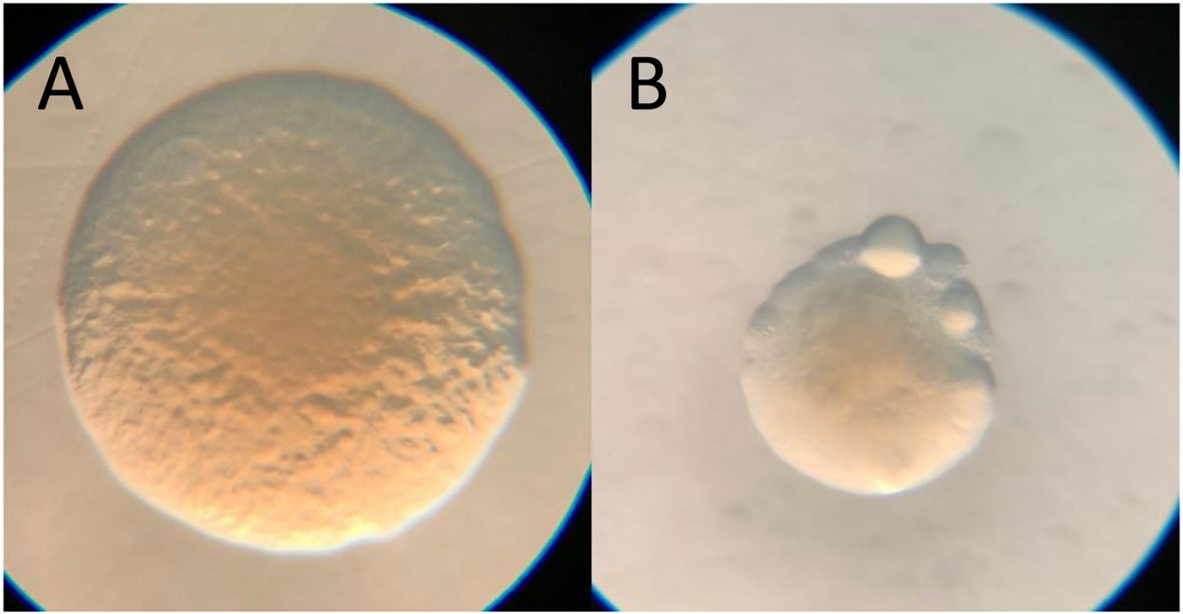
Colonial morphology of untreated *B. cenocepacia* (**A**) and treated with PAS with low dose trimethoprim (**B**). Images collected using a Zeiss Stemi 2000-C stereo microscope at 16X magnification

In the presence of PAS with low trimethoprim, a resurgence of bacterial growth occurred 24 after treatment (Fig. 5B). In comparison to untreated *B. cenocepacia* colonies, which have a regular uniform morphology (Fig. 8 A), colonies formed from PAS treatment were smaller and some were irregular in appearance with a lumpy, cauliflower-like morphology (Fig. 8B). Some of these atypical colonies were tested with the spot assay to determine if they were resistant to KP1 phage. After 24 h, lytic activity was observed for untreated *B. cenocepacia* and *B. cenocepacia* treated with low dose trimethoprim; however, those originally treated with low dose PAS did not show clearing. This indicated untreated *B. cenocepacia* and *B. cenocepacia* treated with just low dose trimethoprim remained susceptible to KP1 while *B. cenocepacia* treated with low dose PAS selected for phage resistance.

## Discussion

*Burkholderia cenocepacia* is an opportunistic pathogen capable of infecting immunocompromised patients, specifically those with cystic fibrosis and chronic granulomatous disease. Due to the high incidence of transmission, “cepacia syndrome”, and resistance to antibiotics, alternative therapies such as bacteriophage therapy are being investigated. The goal of this study was to isolate and characterize a bacteriophage lytic to *Burkholderia cenocepacia* K56-2 and examine its ability to serve as a potential treatment for *B. cenocepacia* infections in CF patients [22, 23].

Although several sources noted isolating *Burkholderia* bacteriophages from onion fields, onion rhizospheres [24] and corn fields [11], we found that raw sewage had a richer supply of bacteriophages, which is where we isolated phage KP1. Electron microscopy of the phage revealed that it has an icosahedral capsid, a tail, and tail fibers. The presence of the tail indicates the phage is in the order *Caudovirales* [25] and possibly in the family *Siphoviridae* family due to the long length of the tail, which is typically 150 nm in length [25]. The *Siphoviridae* family has double stranded, linear DNA comprised of approximately 40–50 kb in length and encoding 55–70 genes [25]. Genome sequencing of phage KP1 DNA indicated it is 52,676 bp. The genome was analyzed for integrase genes seen in other *Burkholderia* phages, but none was detected. A one-step growth curve was performed to understand the replication pattern of the phage within a *Burkholderia cenocepacia* K56-2 culture. Collectively, the lack of integrase genes, replication pattern, and plaque morphology indicate KP1 is a lytic, not temperate, bacteriophage.

Relative to other *B. cenocepacia* lytic phages, KP1 has a similar host range. Seed and Dennis isolated three lytic phages, and two of them infect 4–5 Bcc species [24]. Lynch et al. isolated bacteriophage JG068, which infects Bcc species including *B. cenocepacia*, *B. multivorans, B. stabilis*, and *B. dolosa* [26]. In our spot assay, B. *cenocepacia* K56-2, *B. cenocepacia* J2315, *B. dolosa* AU0645, *B. seminalis* AU0475, *B. cepacia* 25416, *B. lata* HI4002, and *B. arboris* ES263A showed an area of clearing, however, only K56-2, AU0645, AU0475, ES263A and J2315 showed plaques in the plaque assay. Abedon indicates a spot clearing could be due to factors such as toxins [27]. Our hypothesis is that *B. cenocepacia* K56-2 produced a toxin that was in the phage lysate, and this toxin was responsible for the clearing seen in the spot assay for *B. lata* HI4002 and *B. cepacia* 25416. In fact, studies by Yao et al. and Rojas-Rojas, et al. showed that some Bcc species produce a tailocin that is effective against other Bcc species [28, 29]. Perhaps *B. cenocepacia* produced a tailocin that affected the two Bcc species.

Studying bacterial infections in plants can provide important preliminary information about bacterial diseases in animals because of the overlap in hosts’ defense responses. For example, the duckweed model used by Thomson and Dennis showed that it can serve as an alternative infection model for studying Bcc disease and therapeutic strategies [18]. We started with this model as it could serve as a springboard for future studies in more relevant models. Phage KP1 is capable of rescuing duckweed plants from *B. cenocepacia* infection; it promoted significantly higher survival compared to plants infected with the K56-2 control. Because of its effectiveness, we tested the phage in our ASMDM-based CF model that uses synthetic CF sputum [19]. In addition to phage alone, we examined whether PAS could reduce bacterial density in this model. We used the antibiotic trimethoprim, as it has been used to treat *B. cenocepacia* infections in CF patients [30]. Overall, PAS was the most effective treatment against *B. cenocepacia* density. Surprisingly, phage alone did not significantly reduce the bacterial density compared to K56-2 alone. Although there was a reduction with phage only, it was not significantly different from PAS treatment. It is not clear as to why phage alone did not significantly reduce bacterial density, however, we know the phage was active, as indicated by the plaque assays.

A study by Kamal and Dennis showed that treating *B. cenocepacia* with KS14 phage resulted in larger plaque diameters as well as increased phage titers when using sublethal doses of ciprofloxacin, meropenem, and tetracycline in PAS treatment [14]. In our study, KP1 plaque diameters did not change in the presence of PAS indicating that the decrease in *B. cenocepacia* density is not due to the presence of antibiotic influencing the proliferation of KP1. Additionally, phage numbers remained essentially constant over time regardless of whether antibiotic was present. Although Kamal et al. determined increased KS14 phage titers with PAS, there was no increase in phage titers for KS12 suggesting that a change in the titer with PAS is likely dependent on the specific phage used [14].

Because PAS treatments did not increase KP1 phage proliferation, another explanation as to why PAS is more effective than phage alone and antibiotic alone might be connected to a fitness trade-off [31]. Multiple studies have shown that bacteria treated with PAS can develop resistance to phage by modifying cell surface receptors [14, 31–33]. These receptors not only serve as phage attachment sites, but may also confer resistance to antibiotics, serve in the uptake of nutrients, and provide cell wall integrity. When these receptors are altered, fitness costs can occur. These trade-offs can result in deleterious effects for bacteria such that the bacteria become sensitive to antibiotics [14, 31, 32]. Our hypothesis is that *B. cenocepacia* experienced this trade-off which allowed PAS to reduce the bacterial density within the first 24 h.

*B. cenocepacia* rely on a collection of factors that confer resistance to different antibiotics [34, 35]. For this reason, treating *B. cenocepacia* with just antibiotics is not effective, however, exposing *B. cenocepacia* to multiple selective pressures with different mechanisms of action could be beneficial. More specifically, trimethoprim inhibits the synthesis of thymidine, purines, and bacterial DNA by interfering with its ability to synthesize tetrahydrofolic acid. On the other hand, a lytic phage like KP1 kills *B. cenocepacia* by attaching to a cell component, uses the cell’s machinery to make more phage, and then lyses the cell upon release. So, by treating *B. cenocepacia* with both phage and trimethoprim, the bacteria are challenged to adapt rapidly to multiple stressors. This added stress is evident in the morphologies of the colonies that survived PAS treatment with low dose trimethoprim. The decrease in colony size and change in morphology between the untreated and PAS treated *B. cenocepacia* despite the same length of incubation, could indicate that the acquisition of resistance resulted in a reduced growth rate. Decreased growth rate is a fitness cost to acquiring resistance in *Pseudomonas aeruginosa*, another opportunistic pathogen, that was treated with multiple phages targeting different surface receptors [16]. Although the PAS treatment in our study uses phage and antibiotic instead of multiple phages, the idea that a treatment with multiple selective pressures can reduce growth rate as a cost for resistance, is similar to what is observed in this PAS treatment.

This idea that PAS treatment results in decreased fitness could also be supported by the reduction and morphological change of *B. cenocepacia* pellicles formed in ASMDM. PAS has been shown to reduce biofilms in both *Escherichia coli* [15] and *P. aeruginosa* [16], and the morphological change of the pellicle from a fibrous to a nonfibrous appearance could indicate a loss of fitness in *B. cenocepacia*. Pellicle formation has been previously observed with *B. cenocepacia* and is typically regulated via quorum sensing [36]. More specifically the production of the extracellular matrix components in pellicle formation involves the biofilm-stabilizing exopolysaccharide. Additionally, pellicle formation in *Burkholderia glumae* has been found to be regulated both by the same quorum sensing pathway as *B. cenocepacia* as well as by quorum sensing independent genes that are involved in cellulose synthesis [37]. Pellicle formation in these *Burkholderia* species has been shown to be disrupted either by inhibiting the quorum sensing pathway or by the genes coding for the individual extracellular matrix components [36, 37]. Since pellicle morphology was altered in the PAS treated *B. cenocepacia*, a resistance induced fitness cost could have resulted in one or more of the extracellular matrix components not being expressed either via mutations that effect the quorum sensing pathway or the extracellular matrix components. This loss of extracellular matrix components could explain the pellicle morphology change from thick and fibrous to non-fibrous when treated with PAS. Additionally, the change in pellicle morphology could be due to the death of the cells.

Overall, PAS with KP1 and trimethoprim exhibits potential as a treatment for *B. cenocepacia* infection for its ability to be more effective than antibiotic and phage therapy alone in a CF model. Although there was not complete elimination of *B. cenocepacia* using a clinically relevant dose of trimethoprim (i.e., low dose trimethoprim), the surviving cells were mutants that appeared to have gained KP1 resistance at the cost of a normal growth rate and pellicle formation. This decreased fitness of the surviving *B. cenocepacia* in combination with the vast reduction in population, could mean that the infection might be at the level where even the immune system of an immunocompromised individual, like one with CF, could eliminate the remaining bacteria.

## Conclusion

The results of this study suggest that phage-antibiotic therapy is more effective than phage or antibiotic alone in reducing *B. cenocepacia* population in gas-permeable plates. This model might be used to further our understanding of this pathogen, and to develop new ways to eradicate this detrimental infection that, to this day, is still a significant cause of morbidity and mortality in CF patients.

## Data Availability

The data generated and analyzed during the study are available from the corresponding author upon request.
